# Registration of challenging pre-clinical brain images

**DOI:** 10.1016/j.jneumeth.2013.03.015

**Published:** 2013-05-30

**Authors:** William R. Crum, Michel Modo, Anthony C. Vernon, Gareth J. Barker, Steven C.R. Williams

**Affiliations:** a Kings College London, Department of Neuroimaging, Institute of Psychiatry, De Crespigny Park, London SE5 8AF, United Kingdom; b University of Pittsburgh, Department of Radiology & McGowan Institute for Regenerative Medicine, 3025 East Carson St., Pittsburgh, PA 15203, United States; c Kings College London, Department of Psychosis Studies, Institute of Psychiatry, London SE5 8AF, United Kingdom

**Keywords:** Image registration, Chain graph, Magnetic resonance imaging, Parkinson's disease, Stroke

## Abstract

The size and complexity of brain imaging studies in pre-clinical populations are increasing, and automated image analysis pipelines are urgently required. Pre-clinical populations can be subjected to controlled interventions (e.g., targeted lesions), which significantly change the appearance of the brain obtained by imaging. Existing systems for registration (the systematic alignment of scans into a consistent anatomical coordinate system), which assume image similarity to a reference scan, may fail when applied to these images. However, affine registration is a particularly vital pre-processing step for subsequent image analysis which is assumed to be an effective procedure in recent literature describing sophisticated techniques such as manifold learning. Therefore, in this paper, we present an affine registration solution that uses a graphical model of a population to decompose difficult pairwise registrations into a composition of steps using other members of the population. We developed this methodology in the context of a pre-clinical model of stroke in which large, variable hyper-intense lesions significantly impact registration performance. We tested this technique systematically in a simulated human population of brain tumour images before applying it to pre-clinical models of Parkinson's disease and stroke.

## Highlights

• A method for registering highly variable brain image populations.
• Motivated by and applied to pre-clinical images with highly abnormal appearance.
• Tested using simulated images of brains with lesions of varying sizes.
• Applied to Parkinson's disease and stroke model populations.

## Graphical abstract

**Figure:**
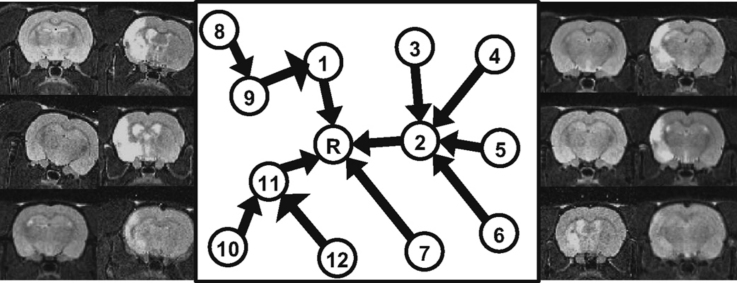

## 1 Introduction

Pre-clinical brain-imaging is increasing in importance, diversity and scale. Non-invasive imaging with Magnetic Resonance Imaging (MRI) facilitates powerful studies that support the three R's (replacement, reduction, refinement) of humane animal experimentation. This is particularly true for serial imaging and in the study of correlations between imaging and histology and how they translate to human studies. However, to extract maximal benefits from imaging studies in terms of scientific gain and the three R's, systematic image processing and analysis are required. Brain imaging studies in humans benefit from an array of automated and semi-automated techniques for analysis, especially in functional MRI (fMRI) and structural MRI (sMRI). One of the most fundamental operations is to register (realign) scans into a common coordinate frame to remove positioning and slicing differences to improve accuracy in inter-subject comparisons (Hill et al., 2001). Registration methods are predicated using brains of similar appearance in the scans; large anatomical or pathological variations can cause standard methods to fail. Images acquired in pre-clinical imaging studies can vary in appearance due to surgical or other interventions and scanning practicalities (especially in legacy data). Our recent experience is that the performance of standard automated affine registration techniques can therefore be severely degraded. However, affine registration is essential to allow for qualitative examination of study images in the same anatomical space and construction of anatomical template images (e.g., means) and is a pre-requisite for mapping localised differences using non-rigid registration. More generally, the development of animal-specific processing pipelines has lagged behind that of human studies, with some notable exceptions (Badea et al., 2012; Clark et al., 2012; Johnson et al., 2007; Lerch et al., 2008). However, the registration of highly variable anatomy in pre-clinical populations remains challenging. Therefore, we have developed a population-based approach to registration in these populations that is successful despite the large variations in appearance observed in typical studies.

### 1.1 Image registration

Image registration is a vital tool in most medical image analysis applications (Crum et al., 2004; Zitova and Flusser, 2003). In the broadest terms, it is used to determine meaningful biological, structural or functional correspondences between medical images (Crum et al., 2003), usually in the form of a coordinate transformation between scans from one or more modalities such as magnetic resonance imaging (MRI), X-ray computed tomography (CT), ultrasound, positron emission tomography (PET), and histopathology. These correspondences allow detailed comparison of anatomy, functional areas (Gholipour et al., 2007) and can help identify imaging biomarkers of disease (Holland et al., 2009; Vernon et al., 2011). An effective registration algorithm is one that reliably establishes plausible and meaningful coordinate transformations within the context of the application. The most common registration task in brain imaging is to align scans of multiple subjects into a single frame of reference for group analysis. This task is so ubiquitous that it is a core component of popular processing software such as Statistical Parametric Mapping (SPM) (Friston et al., 1995) and the FMRIB Software Library (FSL) (Jenkinson et al., 2012).

A measure of correspondence between scans, either direct or surrogate, is required for registration. Direct correspondence measures were originally based on distances between common geometrical features (e.g., landmarks, edges, ridges, surfaces); however, surrogate functions of corresponding voxel-intensities (West et al., 1997) are now widely used in large studies because they are more easily incorporated into automated pipelines. Voxel-similarity measures can model a variety of intensity relationships between registered scans including those that are functional (e.g., correlation ratio) (Roche et al., 1998), or probabilistic (e.g., mutual information (Maes et al., 1997, 2003) or normalised mutual information (Studholme et al., 1999)). The implicit assumption is that maximising the image similarity measure (by adjusting the transformation parameters) maximises the true correspondence between scans. This assumption is violated when image content differs significantly between scans, e.g., because of a large hyper-intense lesion in one scan or because of large non-affine morphological differences. Pre-clinical imaging studies often feature both of these confounding effects. In addition, even when images are similar, naïve automated registration techniques can fail by being trapped in a local minimum during parameter optimisation.

### 1.2 Stroke imaging example

**Fig. 1:**
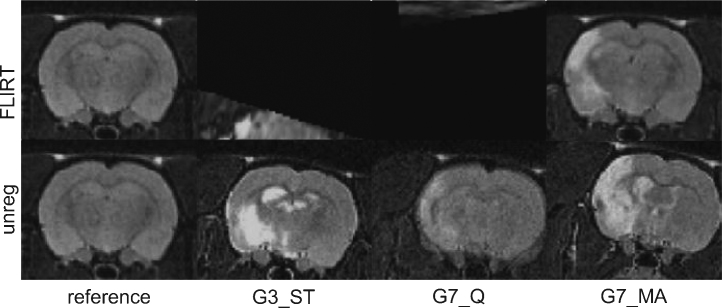
Three examples from the stroke model imaging study demonstrating variations in image positioning, quality and appearance and their effects on FLIRT registration.

This work was initially motivated by our experience with a cohort of 52 scans from a pre-clinical rodent stroke model imaging study, described in detail in Section 3.2.2. Briefly, 39 rats experienced stroke-lesions of varying size and position induced in one hemisphere, and the remaining 13 rats completed a sham procedure. On scanning, the cohort featured variable positioning and image quality combined with highly variable image content (Fig. 1). All scans were rigidly registered to a masked, selected reference using FLIRT – a widely used affine registration software package forming part of FSL. Even using the sophisticated global parameter search available in FLIRT, there were six gross registration failures (defined visually as unequivocal and complete misalignment of the brain) and other more subtle mis-registrations. The gross failure rate might have been reduced by case-by-case optimisation of the standard FLIRT parameters, by providing individual initialisation transformations for poorly positioned scans or by masking individual lesions prior to registration. However, for routine use in neuroscience research, automated and robust solutions are required.

### 1.3 Our contribution

The most commonly used registration techniques explicitly register each image to a reference image. It is well known that registering individual images to a reference image is biased when the reference does not well represent the population and will always be biased to some extent because some subjects will be more similar to the reference than other subjects. An alternative group-wise approach is to implicitly define a reference on a per-study basis in which a measure of (registration) distance from the population is minimised (Bhatia et al., 2007; Learned-Miller, 2006; Studholme and Cardenas, 2004; Twining et al., 2005; Wang et al., 2010a,b). Group-wise registration can be computationally demanding; however, it is not clear that defining such a compromise reference is advantageous when there is large variation in image appearances. However, the situation often arises such that an individual image may differ significantly from the reference, but be more similar to another image in the group. Therefore, from a registration perspective, it makes sense to discover these between-image relationships and traverse the population solving a series of well-defined intermediate registration problems, which can be aggregated to bring all images into the reference space.

Several other researchers have focussed on population-based approaches to registration and analysis, but all have focused on non-rigid procedures. One related technique applied to human brain imaging is manifold learning (Gerber et al., 2010; Hamm et al., 2010; Jia et al., 2010; Wolz et al., 2010), which creates models that efficiently parameterise brain appearance in a relatively low-dimensional space (i.e., low-dimensional space compared to the number of voxels in the brain volume). However, populations with highly variable image appearances due to pathology may not lie on a well-defined low-dimensional manifold; in any case, these techniques assume good affine pre-registration. Another recently reported approach (Wang et al., 2010b) decomposes the group-wise registration problem into a set of smaller problems that are easier to solve. A different approach to population registration is described (Tang et al., 2009) in which a Principal Component Analysis (PCA) is used to generate a series of intermediate templates that are more appropriate for individual members of the population. This method depends on the PCA ability to adequately represent the range of morphological variation in the population; this may not be possible in small populations with large variation. Conceptually, the closest work to ours (Jia et al., 2012) uses a (different) directed graph approach to determine optimal registration paths. However, this approach and other related approaches are focussed on non-rigid morphological (shape) variations rather than pathology and assume that good affine registration already exists for the population. In addition, they have predominantly been applied to shape variation in synthetic and real image populations of normal, or smoothly varying abnormal appearances (e.g., Mild Cognitive Impairment and mild-to-moderate Alzheimer's disease). Hyper- or hypo- intense lesions are generally non-corresponding across images, do not represent shape variation of existing anatomy and can confound even state-of-the-art affine registration techniques.

In this paper we describe a general, robust and efficient framework for affine registration of challenging images – a step that is often taken for granted in the literature. In terms of complexity, our approach lies between the extremes represented by pair-wise and group-wise approaches. It uses a rooted-tree chain graph representation of the image population, in which edge weights are determined by a surrogate distance measure. Nearest-neighbour image pairs on the graph are registered directly, whereas registrations between images that are separated by more than one edge are obtained by transformation composition. We present a general implementation of this approach that uses freely available registration software from the Oxford Centre for Functional MRI of the Brain (www.fmrib.ox.ac.uk/fsl). First we evaluate performance in a model population of human brains, in which lesions of varying size and location were present, and that were subject to known positioning procedures. We then evaluate the technique in two pre-clinical imaging studies: (i) a Parkinson's disease model and (ii) a stroke model.

## 2 Materials and methods

Most image registration techniques in brain imaging studies use measures of image-similarity that are computed from voxel intensities to drive the registration optimisation. The assumption is that image similarity is an acceptable surrogate for biological correspondence and that choosing the transformation that maximises image similarity results in good correspondence properties between brains. Studies in which there are subtle but systematic differences in brain appearance between groups can bias registration. A number of strategies have evolved over the years involving customised templates to try and control for this (Evans et al., 2012). Studies with brains of highly variable and/or abnormal appearance can result in gross registration failures because image similarity is no longer an adequate correspondence substitute. Our hypothesis is that in populations of such brain images, a graph-based approach that directly registers images that are “similar” and infers registrations for images that are “dissimilar” by compositing a series of intermediate registration steps can be effective.

### 2.1 Chain graph representation

**Fig. 2:**
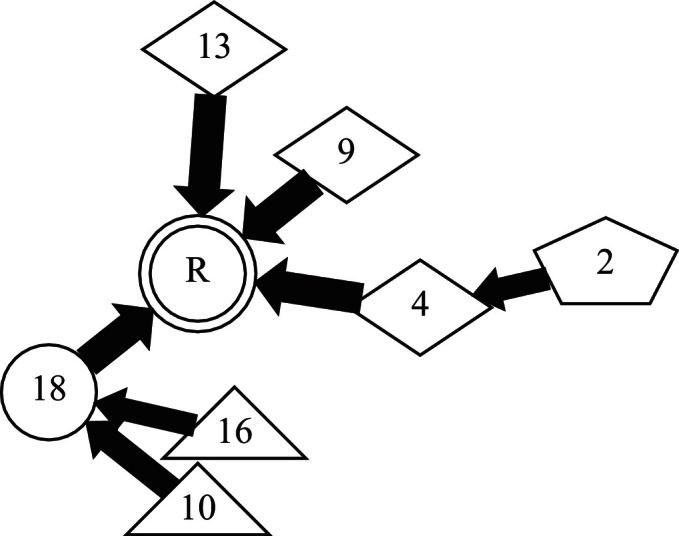
Detail from a rooted-tree chain graph describing image registration over a population. Nodes represent individual images and arrows define pair-wise registrations pointing towards the local reference in each case. The shape of each node encodes the number of registration steps to the global reference, R.

We modelled the image population using a special case of a chain graph representation (Lauritzen and Wermuth, 1989), namely, the rooted tree (or arborescence) variant of the Directed Acyclic Graph (DAG). This model represents a population of registrations as a series of nodes (images) connected by directed edges (registration transformations). A DAG always has directed edges and no loops. The rooted tree has a single root node representing the reference image and there is a single directed path from each node to the root. Any node (image) apart from the reference has one and only one outgoing directed edge connecting it to the next (parent) node and zero or more incoming directed edges from other (child) nodes. Nodes without children are known as leaf nodes. Any image represented as a node on the graph has an unambiguous set of registrations associated with it that are defined by the path across the graph to the root node, and that transforms it into the reference space. Fig. 2 shows an extract from the rooted-tree graph generated for the variable positioning cohort discussed in full in Section 3.1.2.

#### 2.1.1 Distance measure and connectivity

We assume the “distance” between a pair of images can be computed that approximates the between-image differences in appearance and/or spatial configuration and is therefore related to the difficulty of the registration (problems with local minima not withstanding). Image pairs that are more dissimilar or spatially distant will have a larger computed distance. All images are first registered to each other directly in a pair-wise fashion and the pair-wise distance measures are computed resulting in a complete, directed graph with weighted edges. Any initial registrations that fail completely will result in large computed distances. This step scales in computational expense as *n*^2^ (*n* = number of images). However, if pairwise affine registration is fast, then for typical pre-clinical populations of *n* ≪ 100, this step is not computationally restrictive, especially when registrations can be spread over computational cores on a multi-core system. There are many possible parameter optimisation schemes (e.g., from fully exhaustive searches to simplistic steepest-descent methods) that can be used in affine registration; there is a correlation between computing time required for sophisticated or extensive search strategies and the likelihood of finding the optimal registration solution. However, even for the relatively small number of parameters in affine registration of three dimensional MR images, a fully exhaustive search is prohibitively time-consuming and the optimisation landscape is so complex that even sophisticated search strategies can fail to find the global minimum. The performance of any particular optimiser may also be critically dependent on appropriate parameter choices. Our registration technique is designed as an alternative to the FLIRT global optimisation strategy but uses FLIRT for fast individual pair-wise registrations. Therefore, we use FLIRT with the – nosearch option and a custom schedule file to turn off the multi-resolution and global parameter search strategy (see Section 2.3). This ensures a highly localised search strategy and fast operation. For very large populations or instances in which some images are collected after an initial analysis has been performed, the complete graph can be built on a smaller representative sample of m images and the remaining *n* − *m* images can then be registered via the closest corresponding images in the graph. These steps together scale as *m*^2^ + *m*(*n* − *m*) = *mn* ≪ *n*^2^ for *m* ≪ *n*.

**Fig. 3:**
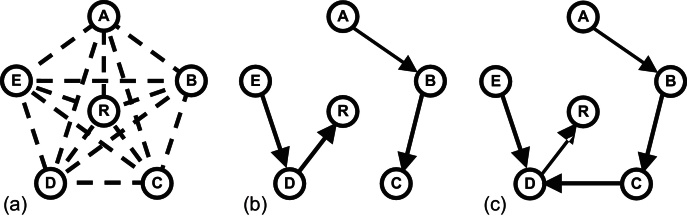
Successful and unsuccessful chain graph construction. (a) Initial comprehensive pair-wise registration, (b) successful rooted-tree chain-graph construction, (c) unsuccessful graph construction – the graph is not connected and there is no registration path from A, B, C to R.

For subsequent chain graph construction to succeed, sufficient pair-wise registrations must succeed so that all scans are connected to all others by one or more pair-wise registrations. Equivalently, we require that the weighted, directed graph is weakly connected after edges corresponding to gross registration failures have been pruned. For example (Fig. 3), for six images A, B, C, D, E, R (= Reference) that are all pair-wise registered to one another, a successful outcome for scans A and R occurs if registrations succeed such that A–B–C–D–R represents a composition of pair-wise registrations that transforms A into the space of R via some intermediate registrations. However, an unsuccessful outcome example would be A–B/C–D–R in which no path exists between A and R because the edge representing the failed registration of C–D has been pruned.

**Fig. 4:**
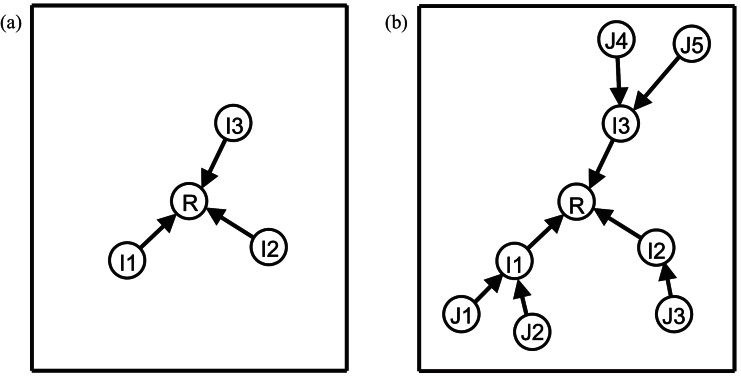
Schematic of graph construction. The rooted tree graph is iteratively built by constructing registration paths (edges) from each scan (node) to the reference, R. (a) Scans for which the reference, R, is amongst the *r*-closest (most-similar) scans are directly connected. The newly connected scans are defined as Tier 1 (= I above). (b) Scans for which the Tier 1 scans are amongst the *r*-closest (most similar) scans are directly connected. The newly connected scans are defined as Tier 2 (= J above).

Successful pruning of the weighted, directed graph (or equivalently, construction of the weakly connected graph), to form the rooted-tree chain graph is key to our approach. This is related to the well-known minimum spanning tree problem for undirected graphs; in directed graphs, this is known as the arborescence problem and can be solved in quadratic time (Chu, 1965). In our case, the distance measures are known to be imperfect, so we aimed to exert stronger control over the branching structure imposed on the graph. Therefore, we investigated two complementary approaches to graph construction that grow the graph iteratively out from the reference node. For large populations, or when scans become available after the initial graph construction, new images can be individually added to the existing graph at low cost. In all of the experiments described in this paper, a single graph construction step was used (Fig. 4).

#### 2.1.2 Rooted-tree graph construction

One image from the population of n images is selected as the nominal reference. This can be arbitrarily specified or defined as the image that is closest to the rest of the population after pairwise registration. Nodes (= images) on the graph are defined by their Tier (the number of edges separating them from the reference node) beginning with the reference (Tier = 0). The distances between all image pairs are sorted and stored and then used to construct the rooted-tree chain graph.

##### 2.1.2.1 Graph construction

The *r* closest images to the reference are connected directly to it and assigned Tier = 1. Then, the pairwise distances between all images and those in the Tier = 1 set are sorted in ascending order. Any unassigned images that have a Tier = 1 image amongst their r-closest neighbours are connected and labelled as Tier = 2. The process is repeated for the next Tier until no more images can be assigned (because any remaining images do not have a peripheral node amongst their r closest images). The value of r is then increased and the process is repeated from Tier = 0 for any remaining unassigned images. An essential part of this algorithm is that connections are made based on the *global* ranking of image distances – that is at each stage, a new image connection to a node is only made if the node is amongst the *r*-most similar images to it in the entire population. The number of incoming edges at each node can vary as *r* is incremented.

The value of *r* must be specified and has a large effect on the graph structure because it determines the number of connections received by each node. Because constructing each graph is computationally fast given pair-wise registrations, we optimise the choice of *r*. Essentially we compute graphs for *r* = 1, 2, 3, 4, …, *n* where *r* = *n* ensures that all nodes are Tier = 1 i.e., each node has a single direct connection to the reference. There are many potential ways to measure graph-fitness. In this work, we have considered simple measures based on the inter-node distance between nodes *i* and *j* (*i* = *j*). We compute the mean and minimum inter-node distances between each node and the reference. We then compute the average of these distance quantities over all nodes. So for a given node, *j*, with *n*_*j*_ nodes inclusive between it and the reference we have:

$$\text{mean}\;\text{inter-node}\;\text{distance}\;\text{of}\;\text{node}\;j:\;{\bar{d}}_{j}=\frac{1}{n_{j}-1}\sum_{k=2}^{n_{j}}d(c_{j}(k),c_{j}(k-1))\text{,}$$

$$\text{min}.\;\text{inter-node}\;\text{distance}\;\text{of}\;\text{node}\;j:\;{\bar{m}}_{j}=\underset{2\leq k\leq n_{j}}{\min}d(c_{j}(k),c_{j}(k-1))\text{,}$$

where *c*_*j*_(*k*) is the *k*’th node between node *j* and the reference, *c*_*j*_(1) = *j*, and *d*(,) returns the distance between two connected nodes. We then define:

$$\text{graph}\;\text{average}\;\text{inter-node}\;\text{distance}:\;d_{\text{mean}}=\sum_{j=1}^{n}{\bar{d}}_{j}$$

$$\text{graph}\;\text{minimum}\;\text{inter-node}\;\text{distance}:\;d_{\min}=\sum_{j=1}^{n}{\bar{m}}_{j}$$

From all candidate graphs we select the one with the smallest *d*_mean_. If more than one of the graphs has the minimum *d*_mean_, we choose the one with smallest *d*_min_.

#### 2.1.3 Rank examples

Construction of the registration graph reduces to two well-known registration paradigms for extreme values of the rank *r*.

**Fig. 5:**
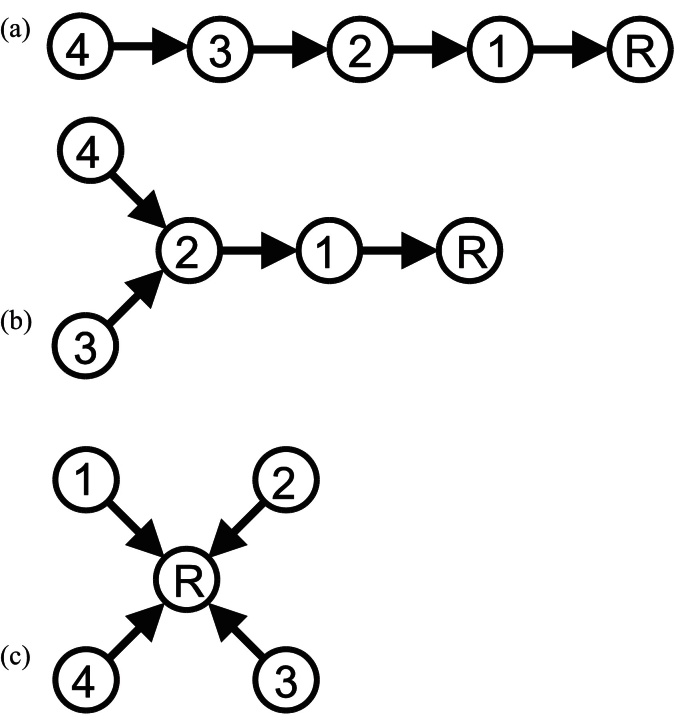
Chain graph structure for extreme values of the rank parameter, *r*, with *n* = 5 images. (a) *r* = 1 gives a serial registration chain if image distances are appropriately ranked, (b) *r* = 1 case with branching structure, (c) *r* = *n* yields a pairwise registration to the reference.

Fig. 5(a) and (b) presents two cases with *r* = 1 where (a) the distances are suitably ranked, yielding a single strand registration chain or (b) a chain with one or more branches. Fig. 5(c) shows the case with *r* = 5 and 5 images where all images are automatically assigned to Tier 1 (because the reference R must be in the top 5 most similar images for each) resulting in traditional pair-wise registration to the reference with no intermediate steps. In practice, the optimised value of r usually lies between these extreme values.

### 2.2 Indirect and direct registration

Once the chain graph is constructed, any image can be transformed into the reference space by taking the uniquely defined path across the graph from that image to the reference. In applications where the image appearance (in terms of intensity, morphology, etc.) is overall very diverse (e.g., our target MRI population of focal stroke lesions), but where images associated with directly connected nodes on the graph are quite similar, generating the net transformation by composition of the intermediates (Indirect Registration) should lead to a good solution. It may be possible to improve results in these applications by performing a Direct registration of each image to the reference using the Indirect Registration transformation as an initialisation.

### 2.3 Implementation

The registration scheduling and graph construction was implemented in Python 2.5.2 (http://python.org/). The program reads a list of images and applies a global threshold to create masks that approximately separate the foreground from the background and define regions of interest on each image. Pairwise registrations were performed, and a table of pair-wise distance measures was computed (i.e., the complete graph). The chain graph was then computed as described in Section 2.1. The graph-construction code is site-independent and is available on request from the lead author. We used the inverse Normalised Mutual Information (NMI) (Studholme et al., 1999) as a surrogate distance measure because it makes the fewest assumptions about intensity relationships between scans and has been found to be robust to overlap variation. A UNIX csh script was then generated to run the Indirect registrations encoded in the chain graph using software from the FMRIB FSL toolkit. Another script was generated to run the Direct registrations using the results of the Indirect Registration as a starting point.

Our current implementation uses the affine registration software FLIRT, a program run from the Linux command-line, which offers considerable options to control its operation. FLIRT already features a sophisticated optimisation strategy designed for volumetric brain registration, which allows for a large capture range in terms of positioning and an efficient parameter search (Jenkinson et al., 2002; Jenkinson and Smith, 2001). However, in this application we overrode this strategy because we have found that it can be confounded by highly variable image appearances and positioning. There are two related options we used to enforce a local parameter search behaviour compared to the standard global search. We (i) invoke the nosearch option, which zeroes the angular search ranges during optimisation, and (ii) we use the schedule option to load a custom schedule file. Schedule files are scripts that allow low-level customisation of FLIRT. Our schedule file (see Appendix A) turns off the multi-resolution and parallel search strategies to ensure that only a fine-scale, local parameter search is performed. Our proposed approach instead uses the population distribution to give a large capture range overall and thus, is robust to appearance variation.

Once the indirect solution has been found by composing registrations across the graph, we assumed that images are approximately aligned with the reference and use a reference brain-mask as a region of interest when the direct registrations were run.

## 3 Experiments

First we quantified the registration error in simulated populations when distinguishing positioning differences from appearance differences was possible. We then applied CHAINS registration to scans from a moderately affected Parkinson's disease model cohort, before studying a challenging population of rat brain images featuring hyper-intense stroke lesions that motivated this work.

### 3.1 Simulated populations

We initially distinguish two extreme registration scenarios: (i) variable appearance = **vA**: in which differences in intensity (i.e., differences due to image acquisition, pathology or scanning artefact, localised variations in shape, etc.) dominate over differences in positioning (i.e., spatial location, orientation, large-scale variations in shape) and (ii) variable positioning = vP: in which differences in spatial configuration dominate over appearance differences. Practical applications should contain a mixture of **vA** and **vP** scenarios, either of which on their own may confound standard registration techniques. To evaluate standard approaches and CHAINS registration in populations dominated by appearance and positioning effects, we used simulated images in which the distribution of spatial transformations to be recovered by registration and the appearance were controlled. We examined three cases: (i) a population of images of diverse appearance in a nominally equivalent spatial configuration corresponding to the ideal **vA** scenario, (ii) a population of morphologically identical images in a range of spatial configurations corresponding to the ideal **vP** scenario, and (iii) a population of images of diverse appearance in a range of spatial configurations, denoted **vAP** (= variable appearance and positioning). Variation in appearance of the images was controlled as was the distribution of spatial configuration to allow for quantitative assessment. We used the T1-weighted isotropic 1 mm × 1 mm × 1 mm voxel BrainWeb digital brain phantom (Collins et al., 1998) for these experiments.

#### 3.1.1 Variable appearance, fixed position population (vA)

**Fig. 6:**
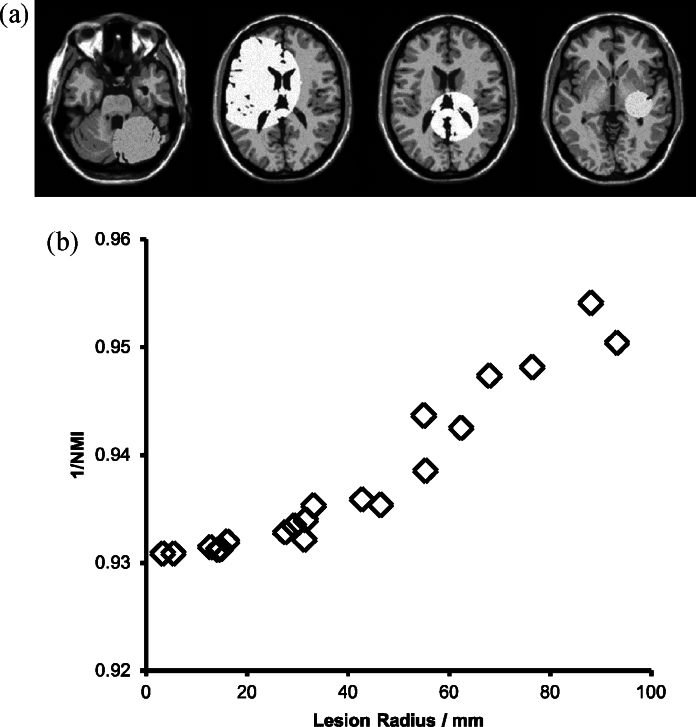
Examples of the **vA** population. Simulated lesions of random sizes and positions change the degree of intensity similarity between the otherwise structurally identical scans. Different slices from the volumes are shown to emphasise lesion variation, but the volumes are positioned identically. (b) The relationship between lesion radius and inverse Normalised Mutual Information in the **vA** population.

To generate the **vA** population, we added independent Rician noise (3% of maximum intensity) and simulated hyper-intense lesions on 20 copies of the phantom as follows. Lesions were simulated as spherical volumes of random radii (uniformly selected from the range 0–100 mm) randomly located in the brain region. A brain mask was applied so that only brain voxels were changed by the lesion; within the spherical lesion volume, all voxels were set to an intensity randomly selected to be between one and two times the mean tissue intensity. One lesion per phantom was applied. Note that this model changes only the voxel intensities and does not simulate any mechanical effect (such as distortion and/or displacement of nearby structures). Application of this model generates a population of images with intensity characteristics that vary due to local tissue properties rather than because of shape or positional changes. Examples of different lesion sizes and intensities are shown in Fig. 6. This population allowed us to assess the extent to which intensity discrepancies can influence registration. All of the images are in perfect alignment a priori, and we explored whether the different registration methods were influenced by the presence of the simulated lesions.

#### 3.1.2 Variable positioning, identical appearance population (vP)

**Fig. 7:**
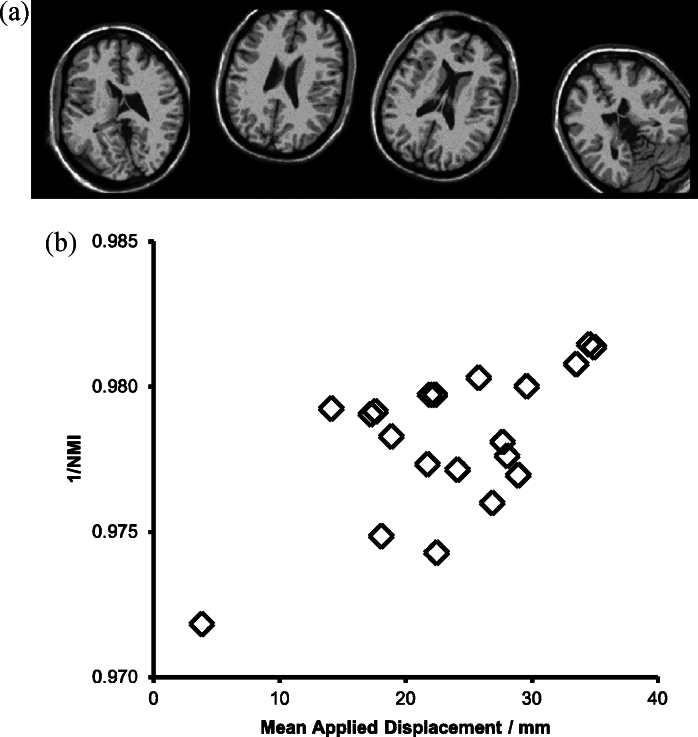
(a) Examples of the **vP** population. Random spatial transformations are applied, but the image content is the same except for Rician noise. Slices from the same 3D position are shown in each case (compare with Fig. 6a, in which different slices are shown). (b) The relationship between mean applied displacement and inverse Normalised Mutual Information for the **vP** population.

To generate the **vP** population, twenty copies of the digital phantom with independent Rician noise (3% of maximum intensity) were transformed with random 9-dof (degree of freedom) transformations. The transformation parameters were selected uniformly from the following ranges: translations: ±20.0 mm, rotations: ±30°, scales: ±0.025). The resulting population consisted of nominally identical images varying only by noise, but with a range of spatial configurations. Examples from the transformed cohort and the relationship between the mean applied displacement and inverse NMI are shown in Fig. 7.

#### 3.1.3 Variable appearance, variable positioning population (vAP)

**Fig. 8:**
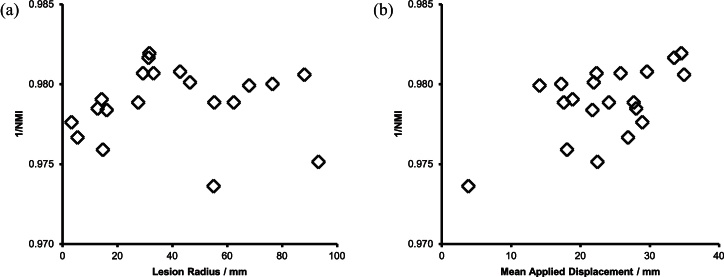
The **vAP** population consists of the images from the **vA** population subjected to the transformations of the **vP** population. (a) The relationship between lesion radius and inverse Normalised Mutual Information in this cohort. (b) The relationship between mean applied displacement and inverse normalised mutual information for this cohort.

To generate the **vAP** population, the transformations generated for the **vP** population were applied to the **vA** population. The relationship between the mean applied displacement, lesion radius and inverse NMI are shown in Fig. 8.

#### 3.1.4 Registration experiments

We ran the following comparisons in each of the populations above using the untransformed, unlesioned brain image as the reference:

• FLIRT-G = FLIRT registrations with default global parameter search options
• FLIRT-L = FLIRT registrations with local parameter search
• CHAINS = our graph-based registration approach
  º CHAINS-I = Indirect CHAINS in which the final transformation is a composition of intermediate transformations obtained by following a path on the DAG.
  º CHAINS-D = Direct CHAINS in which the final transformation is obtained by running a local registration initialised by the CHAINS-I transformation.

For the FLIRT-L and CHAINS registrations, we used a custom schedule file (Section 2.3 and Appendix A) to enforce a local parameter search by turning off the default global optimisation; more details about schedule files are presented in the FLIRT documentation. In all cases, we used Normalised Mutual Information as the registration cost function.

We ran a further experiment to investigate how the choice of rank *r* influenced graph construction and the subsequent registration. The registration of the **vAP** cohort was repeated with the rank, *r*, set to a range of fixed values.

### 3.2 Real populations

We applied the registration techniques to three populations of real images.

#### 3.2.1 Global optimisation test using Parkinson's disease model rat population

**Fig. 9:**
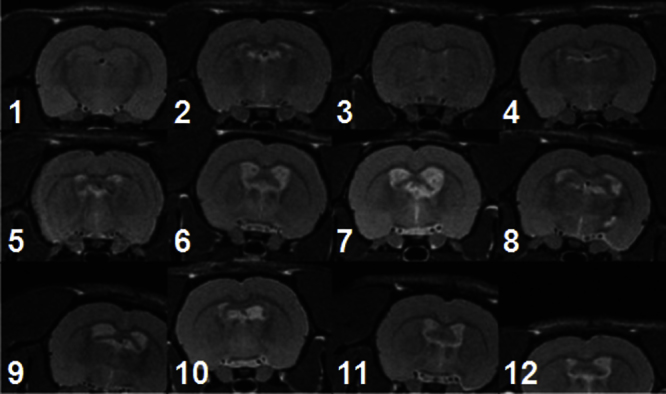
Unregistered MR images from the Parkinson's disease model cohort.

We assessed the optimisation strategy of the CHAINS registration compared to FLIRT-G in a pre-clinical model of Parkinson's disease described in detail elsewhere (Vernon et al., 2011, 2010). Briefly, there were twelve rats included in the study, of which nc = 5 were controls and nd = 7 were in the disease group. All rats underwent the same surgical procedure; controls received an intra-cranial injection of saline and the disease group received an injection of the synthetic proteasome inhibitor Lactacystin into the left-medial forebrain bundle to induce a nigrostriatal lesion. All procedures were in accordance with the UK Animals (Scientific) Procedures Act 1986 and the ethical review process of King's College London. Animals were scanned at 3 time points (1, 3 and 5 weeks post-surgery) in a 7.0 T horizontal small bore magnet (Varian, Palo Alto, CA, USA). MR image acquisition consisted of a multi-echo, multi-slice spin-echo pulse sequence (MEMS) (TR = 4200 ms, TE = 10, 20, 30, 40, 50, 60, 70, 80 ms, 10 averages, matrix size = 192 × 192, FOV = 3.5 cm × 3.5 cm, in plane voxel-size = 0.182 mm × 0.182 mm, number of slices = 50, slice thickness = 0.5 mm, total scanning time per subject = 54 min). Examples from both groups at 3 weeks post-surgery are shown in Fig. 9. Variability in image quality and appearance were apparent but the direct effects of surgical intervention were relatively small. However, the lesion triggers subsequent changes in morphology both proximal and distal to the lesion site (Vernon et al., 2011). A single control subject of good image quality and positioning was reoriented to be symmetric across the mid-line and set as the canonical reference. This made assessment of mis-registration more straight-forward and ensured that registered images could be consistently displayed, even though it may be a sub-optimal choice for the population reference. All subjects were then registered to this reference with 6 dof using FLIRT-G and CHAINS-D to test the population-based optimisation approach used in CHAINS against the pair-wise strategy used in FLIRT.

#### 3.2.2 Graph dependence on selected reference

To assess the dependence of the generated graphs on the choice of reference, we re-ran the graph-construction phase of Section 3.2.1 selecting each individual image as the reference.

#### 3.2.3 Stroke-model rat population

We applied the FLIRT and CHAINS registration approaches in a pre-clinical stroke model (Smith et al., 2012). There were fifty-two rats in the cohort, of which nc = 13 were assigned as the control group and ns = 39 as the stroke group. Stroke lesions were induced using right transient middle cerebral artery occlusion (MCAO); controls underwent the same surgical procedure but without occlusion. The acute lesion manifestation was much more profound than in the Parkinson's disease model described in 3.2.1. All animals were then imaged with MRI prior to any subsequent therapeutic intervention using a horizontal-bore 7 T scanner (Varian, USA). All procedures were in accordance with the UK Animals (Scientific) Procedures Act 1986 and the ethical review process of King's College London. MR image acquisition consisted of a fast spin echo sequence (TR = 3000 ms, Effective TE = 60 ms, RARE factor = 32, averages = 10, matrix size = 128 × 128, FOV = 3 cm × 3 cm, in plane resolution = 0.234 mm × 0.234 mm, number of slices = 45, slice thickness = 0.6 mm, total scanning time per subject = 16 min). Examples of the stroke group images are shown in Fig. 1. Variation in image quality and appearance were apparent. The stroke lesion is of variable size and position (although always appearing on the left side). As in Section 3.2.1, a single sham subject of good image quality was reoriented to be symmetrical across the mid-line and set as the canonical reference. All subjects were then registered to this reference with 6 dof using FLIRT-L, FLIRT-G, CHAINS-I, and CHAINS-D.

## 4 Results

### 4.1 Simulated populations

#### 4.1.1 Variable appearance, fixed position population (vA)

The correct registration to the reference was the identity transformation (because there was no positional variation in this dataset). Therefore, any transformation result that departs from the identity constitutes a registration error. We computed the Residual Displacement Error (RDE) for each case, which was defined as the mean voxel displacement over a brain mask defined on the reference. This value should be equal to 0.0 mm in perfect registration.

**Fig. 10:**
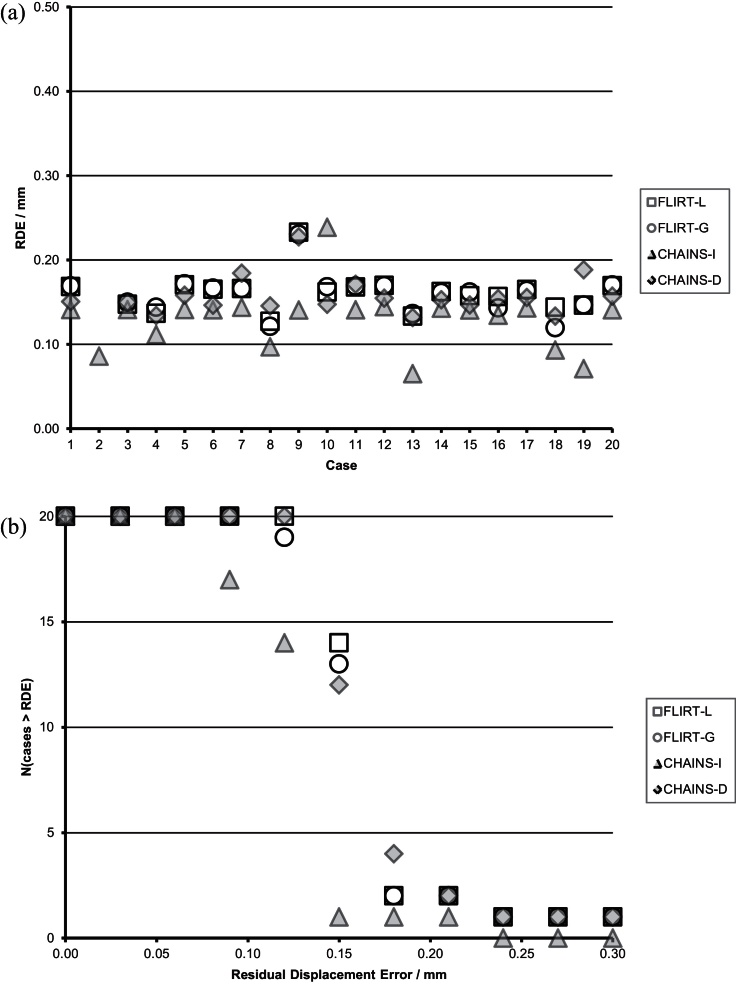
Registration error in the **vA** cohort: (a) for each subject, (b) failures as a function of the RDE failure threshold.

**Fig. 11:**
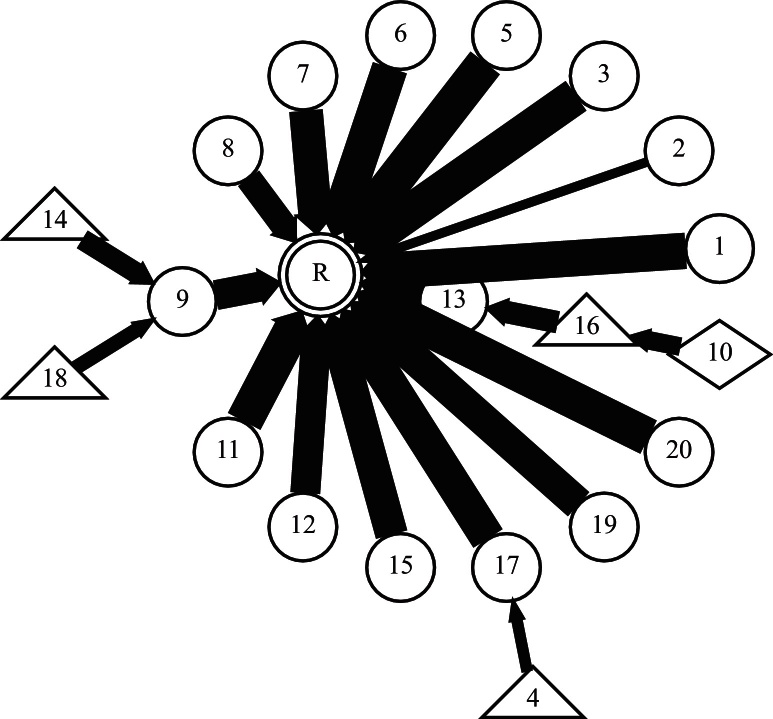
The CHAINS registration graph with *r* = 1 for the **vA** cohort.

Fig. 10(a) summarises the RDE for each member of the **vA** population for the CHAINS methods compared to the FLIRT methods. Fig. 11(b) shows the number of unsuccessful registrations for each method using a threshold of varying RDE as an index of success. No method suffered from any gross registration failures, which we arbitrarily defined as RDE > 1.0 mm; however, there were differences in performance that were measured by RDE. Summary statistics for each method are shown in Table 1.

The CHAINS-I method is the most accurate method over-all; it also had the smallest minimum and maximum error measured per case. This suggests that the detail of graph construction (here with *r* = 1) is an important factor in performance. Image 4 in Fig. 11 corresponds to the largest lesion in Fig. 6 (93.3 mm radius). Interestingly, the second largest lesion (88.1 mm radius) corresponds with Image 2 in Fig. 11; this case was registered directly to the reference but has a large edge-weight (distance) in the graph. The performance for FLIRT-L, FLIRT-G and CHAINS-D were all comparable but less accurate than CHAINS-I; this indicates that for this population, the ability to register through intermediate steps is important for accuracy. Closer inspection of the individual population members revealed that the accuracy difference between CHAINS-D and CHAINS-I is largely driven by a small number of cases (e.g., subjects 2, 13 and 19 in Fig. 10(a)). The CHAINS-D registration was less accurate than the CHAINS-I registration in some instances, even when there was only a single registration step to the reference in both cases. We attributed this to the use of an accurate reference brain-mask in the direct-case, which is used to ensure that only brain features contribute to the registration. In this case, the more accurate mask effectively up-weights the importance of the lesion ROI compared to that defined in the indirect case (in which the simple foreground mask includes contextual information from outside the brain), leading to a less accurate result.

#### 4.1.2 Variable positioning, identical appearance population (vP)

For the **vP** case, the RDE was computed using the residual transformation obtained by composing the known applied transformation (forward) with the transformation recovered (reverse) using each registration method. For perfect registration, the residual transformation should again be the identity matrix giving RDE = 0.0 mm.

**Fig. 12:**
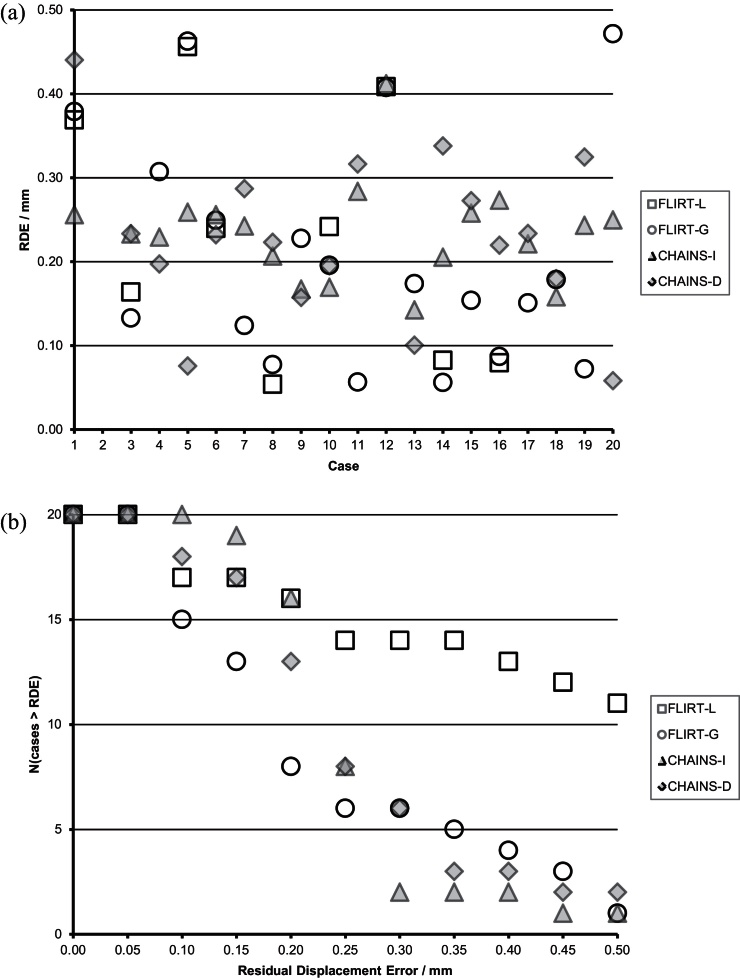
Registration error in the **vP** cohort: (a) for each subject, (b) failures as a function of the RDE failure threshold.

Fig. 12(a) summarises the RDE for each member of the **vP** population in the CHAINS methods compared to the FLIRT methods. Fig. 12(b) presents the number of successful registrations for each method using RDE as an index of success. Summary statistics for each method are shown in Table 2.

**Fig. 13:**
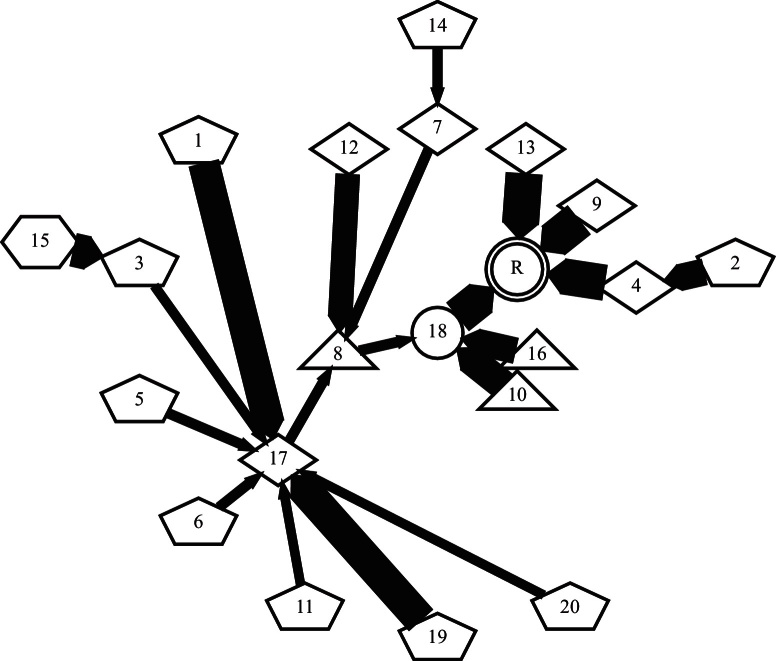
The CHAINS registration graph with *r* = 2 for the **vP** cohort.

This registration scenario is essentially what FLIRT-G was developed to solve; this is reflected by its strong performance. As expected, the performance of FLIRT-L is much worse and 10 cases had an RDE > 1.0 mm and can be considered failed registrations. Neither CHAINS-I nor CHAINS-D were significantly less accurate than FLIRT-G, although it is clear from Fig. 12(a) and (b) that there are some performance differences on individual cases. Fig. 13 presents the registration graph and suggests that the chosen reference (the image with the identity transformation) is not optimal. We consider the choice for the reference further in Section 4.3.2.

#### 4.1.3 Variable appearance, variable positioning population (vAP)

For the **vAP** case, the RDE is computed as for the **vP** case. For perfect registration, the residual transformation should again be the identity matrix giving RDE = 0.0 mm.

**Fig. 14:**
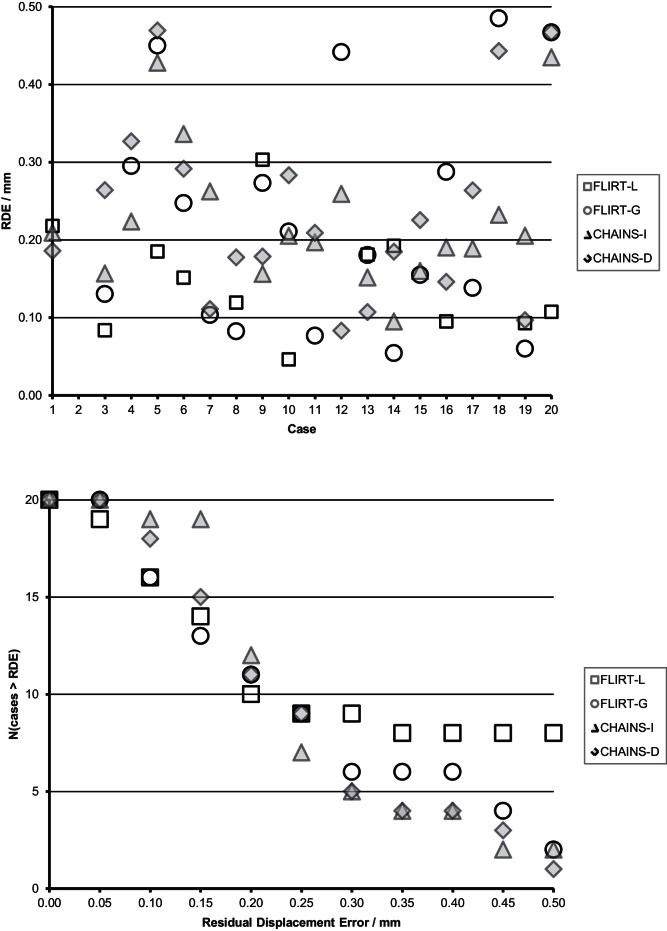
Registration error in the **vAP** cohort: (a) for each subject, (b) failures as a function of the RDE failure threshold.

Fig. 14(a) summarises the RDE for each member of the **vAP** population for the CHAINS methods compared to the FLIRT methods. Fig. 14(b) shows the number of successful registrations for each method using the RDE as an index of success. Summary statistics for each method are presented in Table 3.

**Fig. 15:**
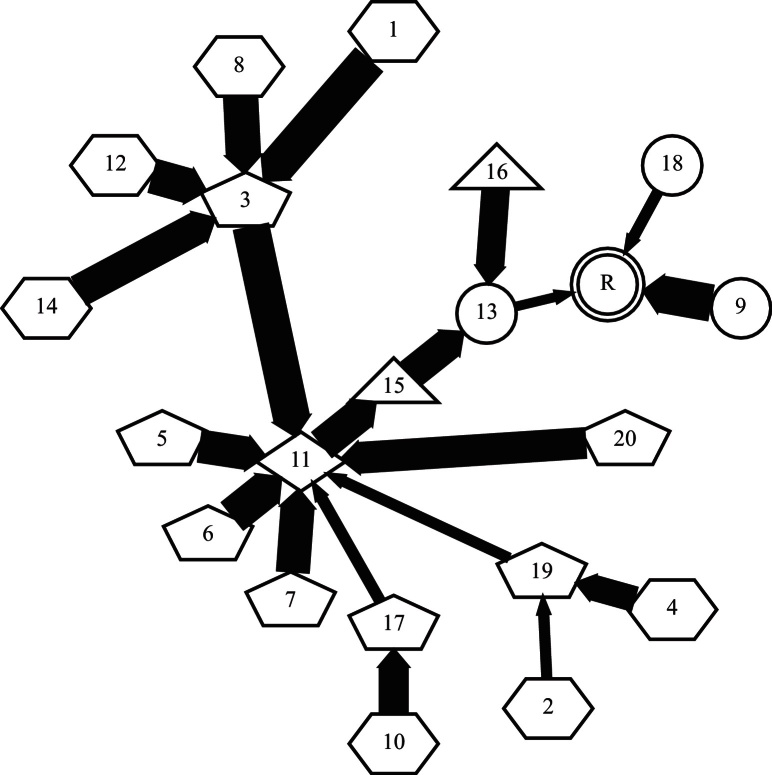
The CHAINS registration graph with *r* = 4 for the **vAP** cohort.

These results are harder to interpret because all methods featured mis-registrations with large RDEs, which skew the results shown in Table 3. Therefore, Table 4 presents the summary statistics for each method after these mis-registrations (defined as RDE > 1.0 mm as before) were removed. It also lists the number of such mis-registrations for each method. FLIRT-L has the lowest mean RDE after 8 mis-registrations have been removed; however, FLIRT-L cannot be considered a reliable technique for general use. Both FLIRT-G and CHAINS-D have only one mis-registration (cases 1 and 2, respectively), though CHAINS-D was more accurate (but not significantly more accurate overall (unpaired, two-tailed t-test, p > 0.5)) and yielded half of the maximum RDE (of successful registrations) observed in FLIRT-G (Fig. 15).

### 4.2 Investigating the graph selection criterion and the rank parameter

First, the **vA**, **vP** and **vAP** registration experiments were rerun with the graph selection made on the basis of the *d*_min_ graph-fitness measure, rather than the default *d*_mean_. This resulted in the same rank/graph choices for the **vA** and **vAP** cohorts as in the original experiments. For the **vP** cohort, using the *d*_min_ graph-fitness measure selected the *r* = 6 graph compared to the *r* = 2 graph using *d*_mean_, but registration accuracies were not significantly different (*p* > 0.3, paired, two-tailed *t*-test).

**Fig. 16:**
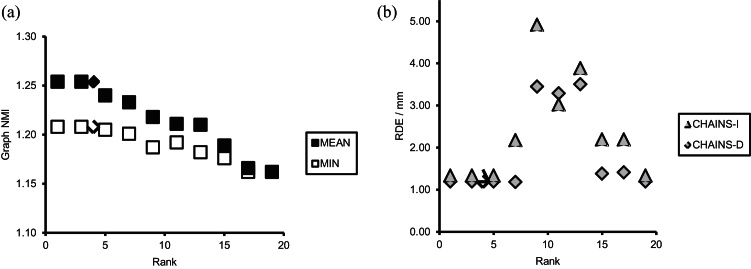
Experiments varying the rank of the CHAINS registration applied to the **vAP** cohort. (a) Graph fitness measures as a function of rank, (b) RDE accuracy measured as a function of rank. The Rank = 4 graph was chosen automatically.

Next, the registrations of the **vAP** cohort were repeated for CHAINS-I and CHAINS-D with the rank, *r*, set to odd numbers in the range 1–19 (odd numbers purely to reduce the number of computations). The fitness measures of the generated graphs presented in Fig. 16(a) show a weak dependence on rank and favour the lower rank cases. The automatic rank selection used in Section 4.1.3 chose rank = 4 for this experiment on the basis of the highest mean NMI and minimum NMI of the resulting graph. The iterative nature of graph construction results in identical output graphs (and therefore identical registration results) for the initial ranks 1–5. Fig. 16(b) also shows the resulting registration accuracies as a function of rank. The RDE is stable and not strongly dependent on rank for small values. In this cohort, an accurate registration result is also found for *r* = 19 that would not have been predicted from the graph fitness measures. It must be emphasised that the behaviour of the registration using different ranks is a complex function of cohort size and population diversity and it is unclear to what extent behaviours will generalise to other cohorts.

### 4.3 Real populations

#### 4.3.1 Global optimisation test using Parkinson's disease model rat population

This experiment does not test registration accuracy *per se*, but assesses the ability of each technique to maximise image similarity, i.e., to find a global optimum of the registration cost function. In the absence of a gold standard for registration, gross mis-registration was defined as cases with unequivocal gross translation and/or rotation of brain structures away from alignment and were judged by careful visual assessment. Using this criterion, we found one extreme failure for FLIRT-G in which the brain was transformed almost completely out of the field-of-view; this failed case, which had NMI = 1.023 for FLIRT-G (failed) and NMI = 1.18 for CHAINS-D (succeeded), was removed from subsequent analysis. We computed the NMI between each of the remaining images and the reference for each registration technique (Table 5) and compared these using paired two-tailed *t*-tests. CHAINS-D resulted in the largest mean and maximum NMI, but was not significantly different (*p* > 0.31) than FLIRT-G overall. FLIRT-L was not significantly different (*p* > 0.87) than FLIRT-G, indicating that for this experiment, the FLIRT-G global search did not result in significantly better optimisation. The CHAINS-D NMI was significantly larger (*p* < 0.025) than the CHAINS-I NMI; this indicates that in this case, the composition of registrations provided by CHAINS-I did not on its own result in the best optimisation, but provided an excellent starting point for the CHAINS-D local search.

**Fig. 17:**
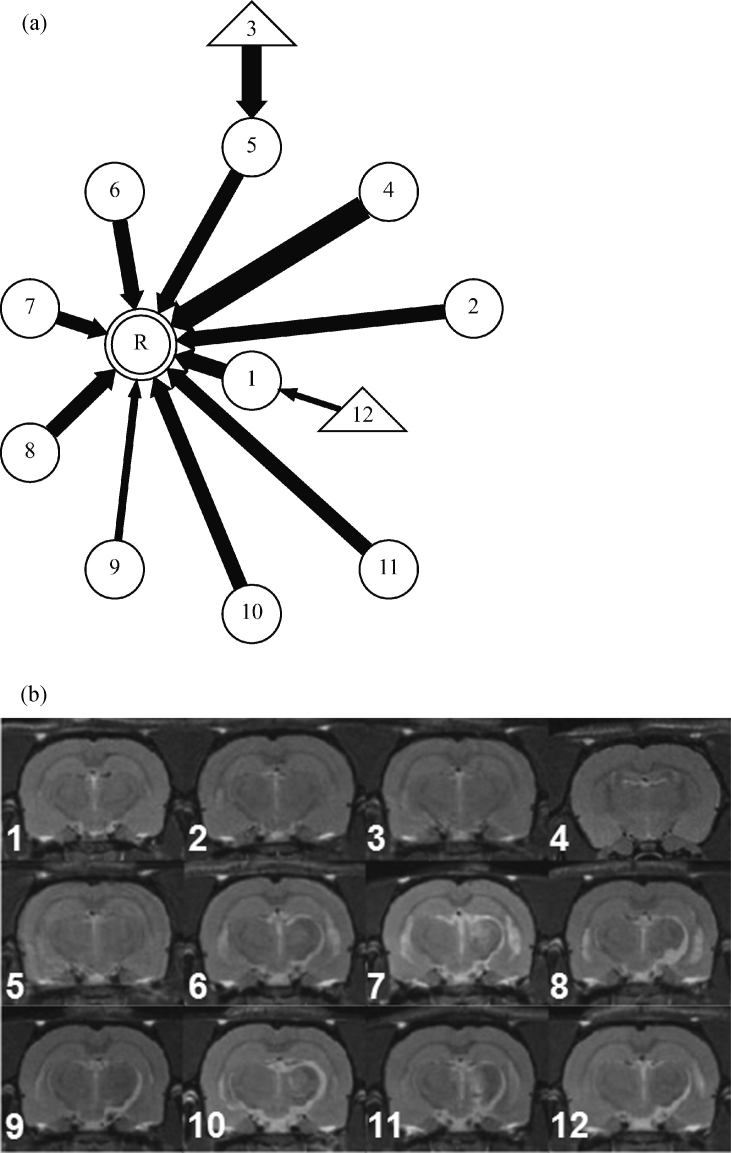
(a) The registration graph for the Parkinson's disease cohort. Note that most scans have simple pair-wise registrations to the reference. The registration of Image 12 by FLIRT-G failed completely. (b) The registered (CHAINS-D) scans corresponding to Fig. 9.

Fig. 17 shows that the graph for this relatively small population defines pair-wise registrations for the majority of scans. The single case that failed with FLIRT-G is number 12 in the figure. This experiment shows that in a population of small contrast variation, CHAINS-D was as good as FLIRT-G in maximising NMI and succeeded in registering one additional case where FLIRT-G failed.

#### 4.3.2 Graph dependence on the selected reference in the Parkinson's model population

The connectivity properties of the graphs generated using each image as the reference are summarised in Table 6. The number of times each pair of images is connected in the set of graphs is shown. It can be observed that there is considerable structure in the connectivity and consistency in the way that many image connections are selected by the majority of graphs constructed. Additionally, other possible image connections are never selected. This suggests that the specifically chosen reference is not crucial to the performance of the technique because the strongest connections are chosen consistently.

#### 4.3.3 Stroke-model rat population

Gross mis-registration was again judged by visual assessment as in Section 4.3.1. This assessment was conducted conservatively to account for the wide variety observed in image appearance and anatomical involvement in the stroke group. The number of mis-registrations for the stroke-group and the entire group are summarised in Table 7.

**Fig. 18:**
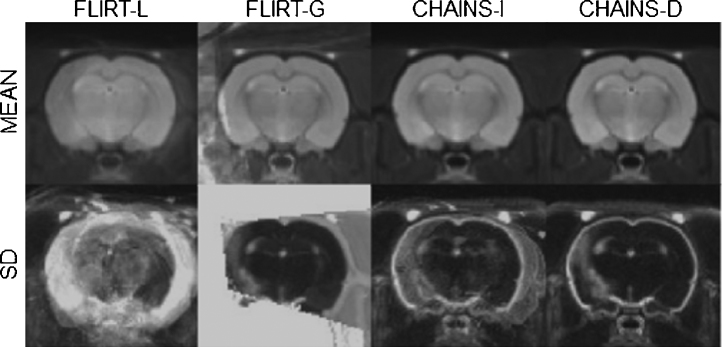
Mean and standard-deviation images of the 52 registered rat brains from the stroke cohort.

**Fig. 19:**
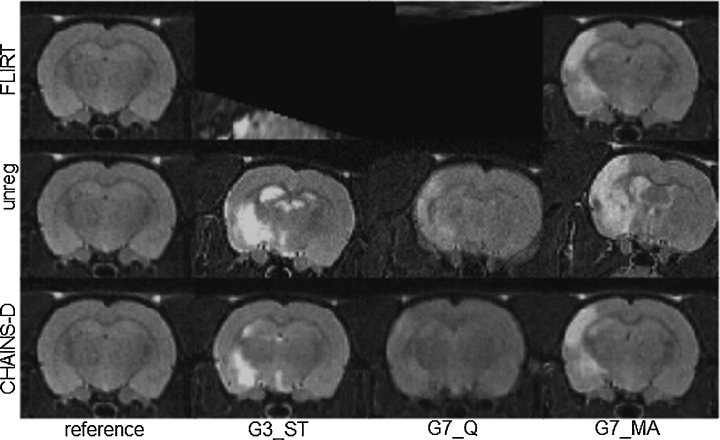
Example scans from the stroke population registered by FLIRT-G and CHAINS-D (6 dof).

We computed mean and standard deviation images of the registered volumes in each case and show an example slice for the four 6-dof cases in Fig. 18. Visual inspection of this figure shows that CHAINS-D produced a sharper mean image and reduced variance maps compared to the other methods. Fig. 19 shows the example results for volumes registered using CHAINS-D that correspond to the images in Fig. 1. Registration graphs for the CHAINS 6-dof and 9-dof cases are shown in Supplementary Figs. 1 and 2.



In the absence of gold-standard registrations to use for comparisons, we used a region overlap criterion as a quantitative measure of relative registration performance. As part of the manual analysis of the original study, regions of interest were drawn around the visible lesion in the 39 stroke scans. After registration using FLIRT-G and CHAINS, we computed the average percentage of lesion voxels that were located inside the brain region drawn on the canonical reference (Table 8).

The overlap percentages should be interpreted with caution; it is possible for two brains to be mis-registered such that the lesion region of interest still coincides with the brain region – just not the correct part of the brain region. A high lesion-brain overlap is necessary, but not sufficient, for good registration. For instance, all six of the registration failures for FLIRT-G were stroke scans with a lesion overlap of 0% indicating complete mis-registration. However, of the three registration failures for CHAINS-I, one was registered from the stroke group but still had a lesion overlap of 91%. Nevertheless, these results support the case for CHAINS as a useful registration strategy for image populations with intensity abnormalities.

## 5 Discussion

In this paper we have presented a practical framework – CHAINS – for solving difficult image registration problems involving populations of diverse appearance and spatial configuration. Specifically, we considered cases in which there were wide variations in appearance due to pathologically or other intensity-based variations occurring together with wide variations in positioning or morphology. CHAINS analyses the population as a whole and uses a composition of well-defined pair-wise registrations to obtain transformations between arbitrary pairs of images. In this way it avoids problems associated with capture-range and poorly defined correspondences. CHAINS is a meta-algorithm in the sense that the registration component (including the distance measure) is essentially arbitrary. CHAINS provides a framework for scheduling, analysing, choosing and assembling transformations obtained using the registration across a population. We used simulated MR images of hyper-intense brain tumours in humans to separate the effects of structure and appearance and show the strengths and limitations of standard registration techniques. In rodent populations modelling Parkinson's disease and stroke, CHAINS performed well despite both image positioning variability and image content variability.

We have used a well-known affine-registration algorithm (FLIRT) as a benchmark in our experiments; however, our work is not meant to imply that FLIRT is poor registration software. Rather, we have found that certain classes of image registration problems, specifically those concerning populations with highly variable appearance, benefit from registration with an alternative optimisation strategy based on learned characteristics of the population. Our motivation was to develop a generic approach that could be used in concert with existing registration techniques and adapted to highly diverse image populations. One important application is spatial normalisation of brain images with focal lesions as in our stroke model exemplar. In human studies, one solution to this problem has been the use of cost function masking (Andersen et al., 2010; Brett et al., 2001; Crinion et al., 2007) in which lesion volumes are explicitly excluded from contributing to registration cost function optimisation. In multiple sclerosis applications in which multiple small lesions are observed, a method for lesion “in-painting” to make MR brain images appear more normal and reduce registration bias has been described (Sdika and Pelletier, 2009). Our approach has the potential to remove bias from the registration of lesion volumes without the need for an explicit mask definition on each volume or alteration of the source images. This could be particularly important in applications in which registration is used to detect subtle remodelling away from the area of pathology as in (Walters et al., 2003).

There are many potential methods for constructing registration graphs and it is not clear which choice of “optimal” graph is best for registration. We investigated the stability of registration results with respect to the rank parameter and found that, at least in our data, our method for choosing rank based on a graph fitness measure also produced the lowest amount of registration error. We also investigated the effect of the reference scan choice on graph connectivity and found that the resulting graphs were highly clustered such that certain pair-wise connections were very likely to occur regardless of the reference chosen. Additionally, a much larger set of possible pair-wise connections never occurred. This suggests that the graph construction method we adopted is likely to be robust to reference choice and automatic rank selection, although more work in this area is warranted. For instance, at present, we do not penalise graphs with long chains, even though they might prove more susceptible to accrued registration error. In addition, a study of connectivity properties across all reference choices (as in Table 6) might form the basis of future graph construction methods.

In Section 1, we mentioned the work of Jia et al. (2012) as being conceptually similar to our approach. They described an image registration technique based on a directed graph approach that optimally selects the reference image and the registration paths from each image to that reference. Practically, the key difference between their work and ours is the implicit assumption in their work that affine-registration is successful before their technique is applied to map shape differences. Their evaluation is of images from normal volunteers and dementia patients; Jia et al. focused on brain shape differences rather than image content differences in their approach. In methodological terms, the graph construction techniques between their method and ours are not directly comparable despite some superficial similarities. An examination of the merits of different graph constructions for registration is an important experiment for the future. In our experiments, to date, we found that the most advantageous aspects of our approach came in tackling content variation rather than shape variation.

Other approaches to image registration where there are correspondence inconsistencies caused by pathology have been reported. One recent area of study has been the problem of registering a normal brain atlas to MR images of brain tumour patients. A common approach has been to use a model of tumour growth to simulate the appearance of pathology in the normal atlas, thus establishing a plausible correspondence (Gooya et al., 2011; Kyriacou et al., 1999; Zacharaki et al., 2009, 2008). A conceptually similar approach is used in Foskey et al. (2005) to resolve correspondence inconsistencies in image-guided prostate radiotherapy that may be caused by the presence of varying amounts of bowel gas. Image deformation is used to shrink the imaged gas pocket to a point, thus restoring the surrounding tissue to a consistent position and morphology. A similar but complementary strategy was used (Gao et al., 2006) to introduce artificial gas pockets in CT radiotherapy planning images to ensure correspondence with daily acquired images. A more subtle challenge is that of tissue contrast changes over time in serial imaging, which may be caused by neurodegenerative processes (Studholme et al., 2006). To address this, a regional measure of Mutual Information has been derived such that local contrast changes are not overwhelmed by global effects when computing the registration. A useful approach for applications in which there are morphological but not topological differences has been reported; in these instance, parametric representations, rather than voxel representations, of the objects of interest are warped together to establish correspondence (Meier and Fisher, 2002). This relies on existing segmentations of the objects to be registered, however, which may limit scalability to large studies.

The adoption of the chain graph is both a potential strength and weakness of our approach. The rooted tree chain graph defines an unambiguous set of registrations between any image in the population and the reference frame. However, if any single registration defined by the graph fails, then all registration paths that include that failed registration will also fail. The likelihood of this situation occurring in practice depends on the size of the image population compared with its diversity and is difficult to quantify. In the experiments reported here, we have found little evidence that this is a significant problem in modest population sizes, even those with rather diverse appearances. In practice, we can also allow the user to tag specific images as “difficult” to ensure they are always defined as leaf nodes on the resulting graph so that they have no incoming edges and other registrations are not dependent on them. The longer term solution is to relax the acyclic condition and allow multiple (but not exhaustive) registration paths between each image and the reference; transformation inconsistency can be used to detect and reject registration failures. This would also add robustness to poor quality or challenging images by introducing redundancy. The increase in algorithmic complexity to achieve this would be considerable and removes the attractive “meta-feature” of the current algorithm.

## 6 Conclusions

With the rapid growth in size and ambition of imaging-based studies, there is a need for registration algorithms that exploit the structure of the imaged population to provide robustness against variations in image structure, appearance and scanner details. CHAINS is one such practical framework that can be easily adapted to use any registration algorithm. We have demonstrated its value in diverse populations of simulated brain lesions, simulated variable positioning, and pre-clinical Parkinson's disease and stroke model populations.

## Figures and Tables

**Table 1:** The Residual Displacement Error (mm) for each registration method tested on the vA population.

vA	FLIRT-L	FLIRT-G	CHAINS-I	CHAINS-D
Mean (mm)	0.18	0.19	0.13	0.18
s.d. (mm)	0.11	0.13	0.04	0.11
Min (mm)	0.13	0.12	0.07	0.13
Max (mm)	0.64	0.73	0.24	0.66

**Table 2:** The Residual Displacement Error (mm) for each registration method tested on the vP population.

vP	FLIRT-L	FLIRT-G	CHAINS-I	CHAINS-D
Mean (mm)	12.00	0.23	0.25	0.26
s.d. (mm)	12.97	0.15	0.09	0.13
Min (mm)	0.05	0.06	0.14	0.06
Max (mm)	33.13	0.54	0.52	0.54

**Table 3:** The Residual Displacement Error (mm) for each registration method tested on the vAP population.

vAP	FLIRT-L	FLIRT-G	CHAINS-I	CHAINS-D
mean (mm)	10.04	88.69	1.33	1.19
s.d. (mm)	13.19	395.46	3.77	4.28
Min (mm)	0.05	0.05	0.10	0.08
Max (mm)	35.54	1768.83	16.26	19.38

**Table 4:** The Residual Displacement Error (mm) for each registration method tested on the vAP population with mis-registrations (defined as RDE > 1.0 mm) removed.

vAP	FLIRT-L	FLIRT-G	CHAINS-I	CHAINS-D
Mean (mm)	0.15	0.27	0.23	0.24
s.d. (mm)	0.07	0.21	0.09	0.12
Min (mm)	0.05	0.05	0.10	0.08
Max (mm)	0.22	0.91	0.43	0.47
*N* (MISREG)	8	1	2	1

**Table 5:** The NMI statistics of the PD model population computed post-registration for four registration techniques.

NMI	FLIRT-L	FLIRT-G	CHAINS-I	CHAINS-D
Mean	1.21	1.21	1.08	1.25
s.d.	0.20	0.09	0.03	0.20
Min	1.04	1.16	1.04	1.16
Max	1.79	1.49	1.17	1.85

**Table 6:**
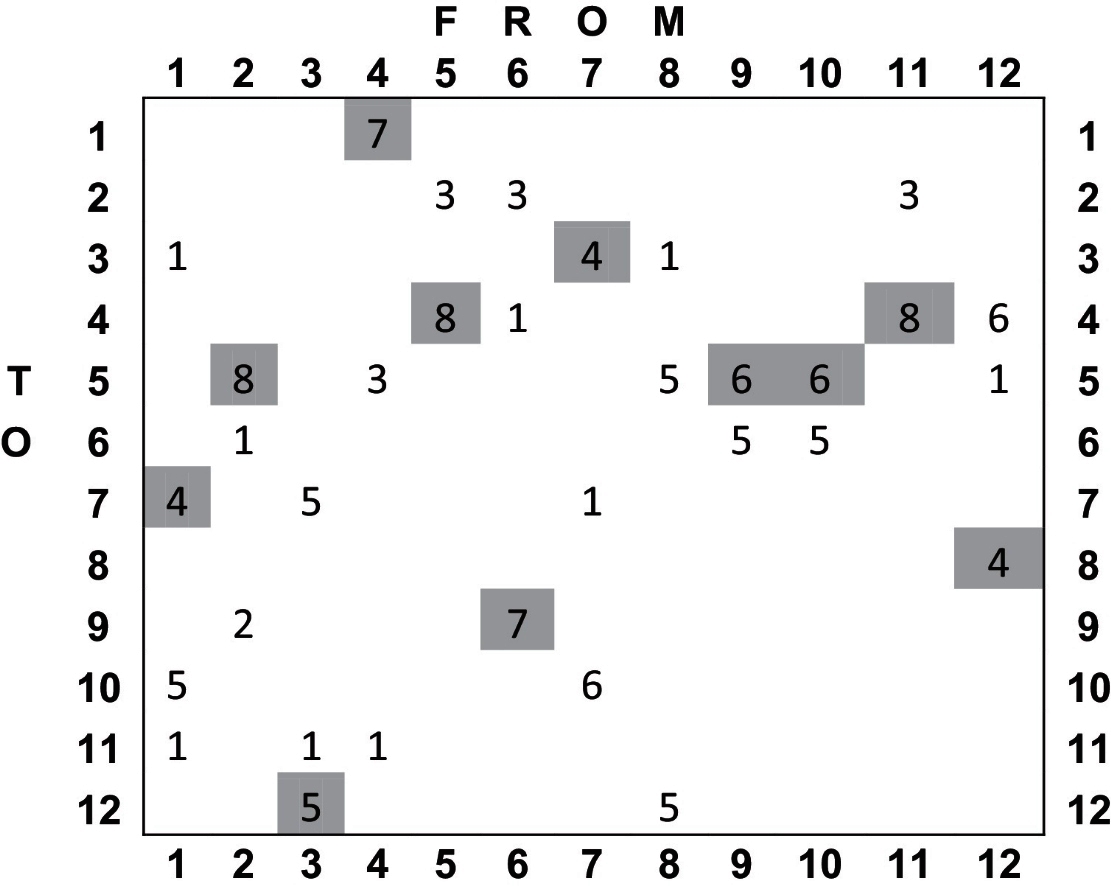
The entries at row *i* and column *j* show the number of times the registration graph featured a connection from image *j* to image *i* when choosing each of the 12 images as the reference. The shaded entries show the connections made for the graph with the automatically chosen reference (Image 8).

**Table 7:** The number of mis-registrations reported in the stroke population.

	*N* (MISREG)	FLIRT-L	FLIRT-G	CHAINS-I	CHAINS-D
6 dof	STROKE	14	6	6	0
6 dof	ALL	17	6	11	0
9 dof	STROKE	15	7	4	0
9 dof	ALL	18	8	4	0

**Table 8:** The average percentage of labelled lesion voxels located within the reference brain region after registration. These results do not include the failed registrations reported in Table 7.

Registration	Lesion overlap %	FLIRT-L	FLIRT-G	CHAINS-I	CHAINS-D
6 dof	STROKE	88.3	90.6	88.9	90.6
9 dof	STROKE	87.0	88.8	87.5	88.7

## Appendix A The custom schedule file used with FLIRT To force a local parameter search

# FLIRT schedule file to run single-resolution local search with no optimisation

# 8 mm scale

setscale 8

clear S

clear P

# 4 mm scale

setscale 4

# 2 mm scale

setscale 2

# 1 mm scale

setscale 1

setoption smoothing 1

setoption boundguess 1

# Set identity transformation

clear UF

setrow UF 1 0 0 0 0 1 0 0 0 0 1 0 0 0 0 1

# Optimise from this position (which includes any user init matrix)

clear U

optimise MAXDOF UF:1 0.0 0.0 0.0 0.0 0.0 0.0 0.0 0.0 0.0 rel 100
