# Supplementary material for: Registration of challenging pre-clinical brain images
Source: J Neurosci Methods. 2013 May 30;216(1):62–77. doi: 10.1016/j.jneumeth.2013.03.015 (PMC3683149; doi:10.1016/j.jneumeth.2013.03.015)
Supplement: Supplementary file 1 [file mmc1.docx]

**Supplementary Data**

Supplementary Fig. 1. The rooted tree chain graph showing registration pathways for the (a) 6dof and (b) 9dof stroke model population. The graph nodes are coded by shape to indicate the number of steps from the reference node. The line-thickness of the edges indicates the pair-wise similarity between connected nodes – thicker = more similar.

Supplementary Fig. 1b.

Supplementary Fig. 1. The rooted tree chain graph showing registration pathways for the (a) 6dof and (b) 9dof stroke model population. The graph nodes are coded by shape to indicate the number of steps from the reference node. The line-thickness of the edges indicates the pair-wise similarity between connected nodes – thicker = more similar.
